# Risk Factors for Nonunion Following Lateral Locked Plating of Distal Femoral Fractures: A Bayesian Analysis of 560 Patients

**DOI:** 10.2106/JBJS.25.00498

**Published:** 2025-11-18

**Authors:** Robert K. Wagner, Stein J. Janssen, Mark Cote, Jacob S. Borgida, Wei Shao Tung, Peter Kloen, Michael J. Weaver, Mitchel B. Harris, Arun Aneja, Thuan V. Ly, the Distal Femur Research Group, Adam N. Musick

**Affiliations:** 1 Department of Orthopaedic Surgery, Massachusetts General Hospital, Boston, Massachusetts; 2 Harvard Medical School Orthopaedic Trauma Initiative, Boston, Massachusetts; 3 Department of Orthopedic Surgery and Sports Medicine, Amsterdam UMC, University of Amsterdam, Amsterdam, The Netherlands; 4 Amsterdam Movement Sciences, Musculoskeletal Health, Amsterdam, The Netherlands; 5 Department of Orthopaedic Surgery, Brigham and Women’s Hospital, Boston, Massachusetts

## Abstract

**Background:**

Lateral locked plating remains an important treatment strategy for distal femoral fractures but has been associated with nonunion rates ranging from 6% to 20%. The objective of this study was to identify factors associated with nonunion following lateral locked plating of distal femoral fractures with use of a Bayesian analysis.

**Methods:**

All consecutive patients ≥18 years of age who were treated with lateral locked plating for a distal femoral fracture at 2 Level-I trauma centers between 2006 and 2024 and who had ≥3 months of follow-up were included. Multivariable Bayesian logistic regression analysis was performed to identify factors associated with nonunion, which was defined as a reoperation to promote healing, and the results are reported as odds ratios (ORs) with 95% credible intervals (CrIs). Probabilities of >95% were considered very strong evidence of an association with nonunion, and probabilities of 90% to 95% were considered strong evidence.

**Results:**

A total of 560 patients (median age, 68 years; 29% male; 90% White; 97% non-Hispanic; 41% with distal periprosthetic fractures) were included. Fifty-four patients (9.6%) underwent reoperation to promote healing. There was very strong evidence that multifragmentary comminution of the metaphysis (versus simple fracture: OR, 2.60; 95% CrI, 0.91 to 8.06), medial cortical comminution of >0 to 25 mm (versus 0 mm: OR, 3.11; 95% CrI, 1.35 to 7.48), and varus (lateral distal femoral angle [LDFA] of ≥84°: OR, 3.04; 95% CrI, 1.46 to 6.51) or valgus (LDFA of ≤78°: OR, 2.42; 95% CrI, 0.96 to 5.99) malalignment increased the odds of nonunion. A screw density of ≤0.60 proximal to the working length reduced the odds of nonunion (versus ≥0.81: OR, 0.40; 95% CrI, 0.16 to 0.95), although the size and certainty of this effect varied in the sensitivity analysis that utilized alternative thresholds. There was strong evidence that obesity increased the odds of nonunion (OR, 1.64; 95% CrI, 0.86 to 3.13) and that intact wedge fractures reduced the odds of nonunion (versus simple fracture: OR, 0.35; 95% CrI, 0.05 to 1.74).

**Conclusions:**

One in 10 patients developed nonunion and underwent reoperation to promote healing. Surgeons should restore coronal plane alignment and may consider augmenting fixation in the presence of multifragmentary comminution. Constructs in which all screw holes proximal to the working length are filled should be avoided, although the optimal configuration remains unclear and depends on other construct characteristics influencing biomechanics. Overall, the small to moderate effect sizes highlight the multifactorial etiology of nonunion following lateral locked plating of distal femoral fractures.

**Level of Evidence:**

Prognostic Level III. See Instructions for Authors for a complete description of levels of evidence.

Lateral locked plating remains an important treatment strategy for distal femoral fractures^1^. However, nonunion rates ranging from 6% to 20% have been reported when lateral locked plating is used in isolation^2-11^. Understanding the factors associated with nonunion following lateral locked plating is important for achieving reliable healing and may guide indications for fixation augmentation (e.g., nail-plate or dual-plate constructs).

Previous studies have identified higher body mass index (BMI), open fractures, and fracture comminution as risk factors for nonunion^2-5,7-9^. Additionally, coronal plane malreduction is a surgeon-controlled variable that has been associated with nonunion, whereas the association of construct characteristics with nonunion has been shown to vary^2-5,7,11-13^.

The current research on this topic relies on frequentist statistics (e.g., p values based on null hypothesis testing and confidence intervals), which can be challenging to apply to clinical decision-making in which estimates of probability are more helpful. Bayesian analysis provides the direct probabilities that an effect is present and that it exceeds clinically relevant thresholds, and allows better interpretation of effect sizes and the certainty of effects. As such, Bayesian analysis is increasingly being used, including in the orthopaedic trauma literature^14-17^. The objective of this study was to identify factors associated with nonunion following lateral locked plating of distal femoral fractures with use of Bayesian analysis.

## Materials and Methods

### Patients

After institutional review board approval, all consecutive patients ≥18 years old with a distal femoral fracture (AO/OTA^18^ 33A or 33C) treated with open reduction and internal fixation (ORIF) or minimally invasive plate osteosynthesis (MIPO) using lateral locked plating between January 2006 and June 2024 were identified from the institutional research database of 2 Level-I trauma centers with use of Current Procedural Terminology (CPT) codes 27511 and 27513. The 7 exclusion criteria were (1) pathological fracture, (2) <3 months of clinical or radiographic follow-up (unless the patient had healed and was discharged from follow-up, or had experienced an outcome event, before 3 months), (3) index surgery was a revision surgery, (4) staged treatment (with the exception of external fixation), (5) bilateral distal femoral fractures, (6) prior below-the-knee amputation, and (7) paraplegia. All lateral locking plates were manufactured by DePuy Synthes and included the Locking Compression Plate with Less Invasive Stabilization System instrumentation (LCP LISS) (n = 233; 42%), LISS Distal Femur (LISS DF) (n = 223; 40%), Variable Angle (VA)-LCP Curved Condylar (n = 82; 15%), and LCP Condylar (n = 22; 3.9%). Six patients who were treated with another lateral locking plate system were excluded. Ninety-two (14%) of the otherwise eligible patients (n = 652) were excluded due to insufficient follow-up.

### Explanatory Variables

**Fig. 1:**
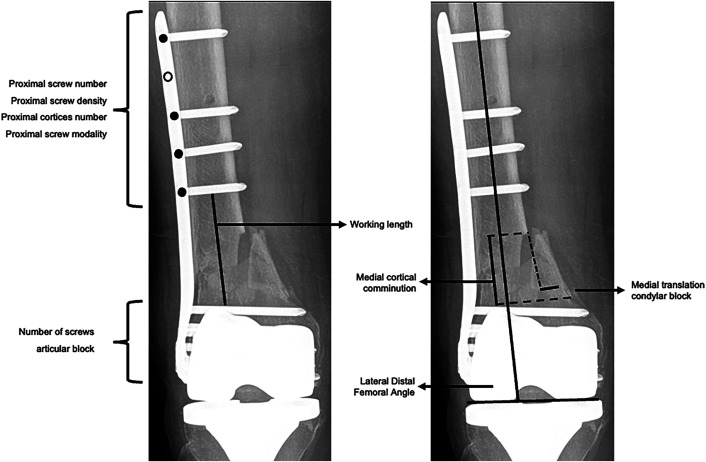
Radiographic fracture, fixation construct, and fracture reduction characteristics.

The baseline patient and injury characteristics included age, sex, race, ethnicity, diabetes mellitus, tobacco use, obesity (BMI ≥30 kg/m^2^), American Society of Anesthesiologists (ASA) classification, metaphyseal fracture pattern based on the AO/OTA classification^18^ (simple, intact wedge, or multifragmentary or fragmentary wedge [henceforth referred to as multifragmentary]), intra-articular fracture, the presence of ipsilateral implants, Gustilo-Anderson classification^19^, associated vascular injury, and the length of medial cortical comminution (0, >0 to 25, or ≥26 mm). Race and ethnicity data were extracted from the institutional research database and reported descriptively. Medial cortical comminution was the distance between the proximal and distal intact segments of the medial cortex and was measured parallel to the femoral shaft axis. Treatment characteristics included plate metal, plate type (LCP LISS and LISS DF versus VA-LCP Curved Condylar and LCP Condylar), plate length (in mm), plate working length (in mm), the number of screws proximal to the working length, proximal screw density (≤0.60, 0.61 to 0.80, or ≥0.81), proximal screw modality (all-locking versus hybrid or non-locking), the number of cortices captured by the proximal screws, the number of screws in the condylar block, the medial translation of the condylar block (in mm), and the lateral distal femoral angle (LDFA; ≤78°, 79° to 83°, or ≥84°). Working length was the distance between the nearest screws proximal and distal to the fracture^3^. Proximal screw density was the number of proximal screws divided by the number of screw holes proximal to the working length. Medial translation of the condylar block was the distance between lines drawn tangential to the medial cortices of the distal and proximal fracture segments^5,11^. Explanatory variables were collected from electronic medical records by orthopaedic trauma research fellows. All radiographic assessments were performed by a postdoctoral orthopaedic trauma research fellow with use of standardized assessments (Fig. 1) following training by 2 fellowship-trained orthopaedic trauma surgeons. The LDFA, medial translation, and medial cortical comminution were assessed on post-reduction anteroposterior radiographs; a second author independently assessed these measures in a randomly selected subset of 10% of the patients in order to determine interobserver reliability. Length measurements were calibrated using the distance between plate screw holes^5,11,20^.

### Outcome

The primary outcome was nonunion, defined as a reoperation to promote fracture-healing, as documented in the operative notes of the treating surgeon^5,20^. This included unplanned reoperations such as revision fixation or bone-grafting to promote fracture-healing and early revision for implant failure^5,20^.

### Statistical Analysis

Descriptive statistics are presented as frequencies and percentages for categorical variables and as medians and interquartile ranges (IQRs) for continuous variables. Medial cortical comminution, proximal screw density, and the LDFA were categorized on the basis of a nonlinear relationship visualized on locally weighted scatterplots. Multivariable Bayesian logistic regression was used to determine the independent association of each explanatory variable with nonunion. Explanatory variables with an 80% credible interval (CrI; the range of values within which the odds ratio [OR] is expected to fall with the specified probability) that did not cross 1.00 in the univariate regression were included in the multivariable regression. A “prior” for the intercept (i.e., prior knowledge about the baseline risk for nonunion) was modeled by assuming a baseline risk of 9% for nonunion, with a plausible range of 3.5% to 21%. The final model was adjusted for the number of days between each patient’s index surgery and the earliest surgical date in the dataset. All models included a weakly informative prior (mean, 0; standard deviation, 1) for the effect of explanatory variables on nonunion, in order to reflect uncertainty regarding the size and direction of effects within a plausible range of values. The results of the multivariable analysis are reported as ORs with 95% CrIs, and the probability that each OR exceeded or fell below specific thresholds (pOR) was calculated. Probabilities of >95% were considered very strong evidence; 90% to 95%, strong evidence; 75% to 89%, moderate evidence; and 50% to 74%, weak evidence. Less than 3% of the combined data points for variables included in the final model were missing and were imputed prior to performing the multivariable analysis using multivariate imputation by chained equations^21^. Two post hoc sensitivity analyses were performed. The first sensitivity analysis included patients with ≥5.5 months of follow-up (since the exact timing of the 6-month follow-up may vary). The second analysis included alternative screw density categories. Model diagnostics indicated good Markov chain Monte Carlo performance and convergence, and posterior predictive checks demonstrated a good fit with the observed nonunion rate for each analysis on each imputed dataset. To aid interpretation of the Bayesian analysis, a multivariable logistic regression analysis was performed, and the results were reported as ORs with 95% confidence intervals (CIs). Intraclass correlation coefficients (ICCs) with 95% CIs were calculated to assess interobserver reliability with use of a single-rater, absolute agreement, 2-way random-effects model. All statistical analyses were performed using R (version 4.3.0; R Foundation for Statistical Computing) and the packages mice^21^, rstanarm^22^, broom^23^, and tidybayes^24^.

## Results

### Baseline Characteristics

A total of 560 patients were included. The median age was 68 years (IQR, 59 to 77 years), 29% of the patients were male, 90% were White, and 97% were non-Hispanic. Of the included patients, 87 (16%) were active tobacco users and 219 (43%) were obese at the time of injury (Table 1). There were 350 (63%) fractures with multifragmentary comminution, 237 (42%) intra-articular fractures, 231 (41%) distal periprosthetic fractures, and 58 (10%) open fractures. Fifty-four patients (9.6%) underwent reoperation to promote fracture-healing. The median clinical or radiographic follow-up was 15 months (IQR, 7 to 51 months). The ICC was 0.83 (95% CI, 0.73 to 0.90) for the LDFA, 0.81 (95% CI, 0.70 to 0.89) for medial translation, and 0.96 (95% CI, 0.92 to 0.98) for medial cortical comminution.

**Table 1:** Patient and Injury Characteristics with Univariate Bayesian Logistic Regression Results*

	All (N = 560)	No Nonunion (N = 506)	Nonunion (N = 54)	OR (80% CrI)
Patient characteristic				
Age *(yr)*	68 (59-77)	68 (59-77)	68 (55-74)	0.99 (0.98-1.00)†‡
Male *(no. [%])*	160 (29%)	148 (29%)	12 (22%)	0.70 (0.45-1.06)
Diabetes mellitus *(no. [%])*	129 (23%)	117 (23%)	12 (22%)	0.94 (0.60-1.43)
Tobacco use§ *(no. [%])*	87 (16%)	78 (16%)	9 (17%)	1.06 (0.64-1.68)
Obese§,# *(no. [%])*	219 (43%)	191 (42%)	28 (55%)	1.65 (1.14-2.40)‡
ASA classification *(no. [%])*				
I-II	227 (41%)	205 (41%)	22 (41%)	Reference
III-IV	333 (59%)	301 (59%)	32 (59%)	1.00 (0.70-1.44)
Injury characteristic				
Metaphyseal fracture pattern *(no. [%])*				
Simple	139 (25%)	134 (26%)	5 (9.3%)	Reference
Intact wedge	71 (13%)	69 (14%)	2 (3.7%)	0.72 (0.24-1.85)
Multifragmentary or fragmentary wedge	350 (63%)	303 (60%)	47 (87%)	3.82 (2.22-6.98)‡
Intra-articular fracture *(no. [%])*	237 (42%)	209 (41%)	28 (52%)	1.51 (1.06-2.15)‡
Periprosthetic, distal *(no. [%])*	231 (41%)	210 (42%)	21 (39%)	0.90 (0.62-1.30)
Periprosthetic or peri-implant, proximal *(no. [%])*	94 (17%)	89 (18%)	5 (9.3%)	0.48 (0.25-0.84)‡
Gustilo-Anderson type *(no. [%])*				
Closed	502 (90%)	457 (90%)	45 (83%)	Reference
I-II	24 (4.3%)	21 (4.2%)	3 (5.6%)	1.30 (0.51-2.79)
III	34 (6.1%)	28 (5.5%)	6 (11%)	2.03 (1.07-3.66)‡
Medial cortical comminution *(no. [%])*				
0 mm	268 (48%)	254 (50%)	14 (26%)	Reference
>0-25 mm	102 (18%)	82 (16%)	20 (37%)	4.09 (2.58-6.55)‡
≥26 mm	190 (34%)	170 (34%)	20 (37%)	2.01 (1.29-3.17)‡
Vascular injury *(no. [%])*	10 (1.8%)	8 (1.6%)	2 (3.7%)	2.03 (0.60-5.52)

* Values are given as the count, with the percentage in parentheses, or as the median, with the interquartile range in parentheses. OR = odds ratio, CrI = credible interval, ASA = American Society of Anesthesiologists.

† OR per 1-year increase. The upper bound of the 80% CrI was 0.998 before rounding.

‡ Variable for which the 80% CrI did not cross 1.00.

§ Data were missing for tobacco use (n = 7; 1.3%) and obese (n = 54; 9.6%). The percentages given for tobacco use and obese in the table are based on the number of patients with available data.

# Body mass index of ≥30 kg/m^2^.

### Univariate Analysis

The results of the univariate Bayesian logistic regression analysis are displayed in Tables 1 and 2.

**Table 2:** Treatment Characteristics with Univariate Bayesian Logistic Regression Results*

Treatment Characteristic	All (N = 560)	No Nonunion (N = 506)	Nonunion (N = 54)	OR (80% CrI)
Plate metal† *(no. [%])*				
Stainless steel	139 (25%)	120 (24%)	19 (37%)	Reference
Titanium	410 (75%)	377 (76%)	33 (63%)	0.61 (0.42-0.90)‡
Plate type *(no. [%])*				
LCP LISS or LISS DF	456 (81%)	416 (82%)	40 (74%)	Reference
VA-LCP Curved Condylar or LCP Condylar	104 (19%)	90 (18%)	14 (26%)	1.56 (1.02-2.35)‡
Plate length *(mm)*	236 (236-276)	236 (236-276)	236 (236-259)	0.99 (0.97-1.01)§
Plate working length *(mm)*	58 (38-79)	58 (38-79)	61 (38-87)	1.02 (0.97-1.07)§
No. of proximal screws	5 (4-6)	5 (4-6)	5 (4-6)	0.98 (0.85-1.11)
Proximal screw density *(no. [%])*				
≥0.81	177 (32%)	157 (31%)	20 (37%)	Reference
0.61-0.80	201 (36%)	178 (35%)	23 (43%)	1.03 (0.68-1.54)
≤0.60	182 (33%)	171 (34%)	11 (20%)	0.52 (0.32-0.84)‡
No. of proximal cortices	8 (6-9)	8 (6-9)	8 (5-8)	0.95 (0.87-1.03)
Proximal screws, all locking *(no. [%])*	485 (87%)	443 (88%)	42 (78%)	0.52 (0.33-0.83)‡
No. of distal screws, condylar block	6 (6-7)	6 (6-7)	6 (5-7)	0.90 (0.74-1.11)
Medial translation of the condylar block† *(mm)*	3.0 (0.0-6.5)	3.0 (0.0-6.5)	4.1 (1.6-7.5)	1.32 (1.07-1.61)‡#
LDFA† *(no. [%])*				
79°-83°	250 (48%)	238 (50%)	12 (27%)	Reference
≤78°	79 (15%)	70 (15%)	9 (20%)	2.31 (1.29-4.06)‡
≥84°	190 (37%)	167 (35%)	23 (52%)	2.52 (1.62-4.00)‡

* Values are given as the count, with the percentage in parentheses, or as the median, with the interquartile range in parentheses. OR = odds ratio, CrI = credible interval, LCP = locking compression plate, LISS = less invasive stabilization system, DF = distal femur, VA = variable angle, LDFA = lateral distal femoral angle.

† Data were missing for plate metal (n = 11; 2.0%), medial translation of condylar block (n = 1; 0.2%), and LDFA (n = 41; 7.3%). The percentages given in the table for plate metal and LDFA are based on the number of patients with available data.

‡ Variable for which the 80% CrI did not cross 1.00.

§ OR per 10-mm increase.

# OR per 5 mm.

### Multivariable Analysis

There was very strong evidence that multifragmentary metaphyseal fracture patterns (versus simple: OR, 2.60; 95% CrI, 0.91 to 8.06), medial cortical comminution of >0 to 25 mm (versus 0 mm: OR, 3.11; 95% CrI, 1.35 to 7.48), and coronal plane malalignment with an LDFA of ≥84° (OR, 3.04; 95% CrI, 1.46 to 6.51) or ≤78° (OR, 2.42; 95% CrI, 0.96 to 5.99) were associated with increased odds of nonunion and that a screw density of ≤0.60 proximal to the working length (versus ≥0.81: OR, 0.40; 95% CrI, 0.16 to 0.95) was associated with reduced odds (Table 3). Further, there was strong evidence that obesity was associated with increased odds of nonunion (OR, 1.64; 95% CrI, 0.86 to 3.13) and that intact wedge fractures (versus simple: OR, 0.35; 95% CrI, 0.05 to 1.74) were associated with reduced odds. The results of the multivariable frequentist logistic regression are displayed in Appendix 1.

**Table 3:** Results of Multivariable Bayesian Logistic Regression Analysis for Risk Factors for Nonunion*

Characteristic	OR (95% CrI)	Probability That the Odds of Nonunion Are Above or Below the Stated Threshold					
		<0.5	<0.67	<1	>1	>1.5	>2.0
Patient or fracture characteristic							
Age, 1-year increase	0.99 (0.97-1.02)	<1%	<1%	77%	23%	<1%	<1%
Obese†	1.64 (0.86-3.13)	<1%	<1%	7%	93%	60%	27%
Metaphyseal fracture pattern (reference: simple)							
Intact wedge	0.35 (0.05-1.74)	66%	78%	90%	10%	4%	2%
Multifragmentary or fragmentary wedge	2.60 (0.91-8.06)	<1%	<1%	4%	96%	85%	69%
Intra-articular fracture	1.06 (0.53-2.11)	2%	10%	43%	57%	16%	4%
Periprosthetic or peri-implant, proximal	0.56 (0.18-1.49)	41%	63%	87%	13%	2%	<1%
Gustilo-Anderson type (reference: closed)							
I-II	1.27 (0.26-4.50)	11%	20%	37%	63%	41%	25%
III	1.52 (0.46-4.47)	3%	9%	24%	76%	51%	31%
Medial cortical comminution (reference: 0 mm)							
>0-25 mm	3.11 (1.35-7.48)	<1%	<1%	<1%	>99%	96%	85%
≥26 mm	1.18 (0.52-2.81)	2%	9%	35%	65%	29%	12%
Treatment characteristic							
Titanium plate (reference: stainless steel)	0.62 (0.28-1.42)	30%	57%	87%	13%	2%	<1%
VA-LCP Curved Condylar or LCP Condylar (reference: LCP LISS or LISS DF)	0.74 (0.19-2.78)	28%	44%	67%	33%	15%	7%
Proximal screws, all locking (reference: other modalities)	0.52 (0.15-1.78)	48%	66%	85%	15%	5%	2%
Proximal screw density (reference: ≥0.81)							
0.61-0.80	0.77 (0.38-1.55)	11%	35%	77%	23%	3%	<1%
≤0.60	0.40 (0.16-0.95)	70%	88%	98%	2%	<1%	<1%
Medial translation of the condylar block, 5-mm increase	1.10 (0.77-1.57)	<1%	<1%	30%	70%	4%	<1%
LDFA (reference: 79°-83°)							
≤78°	2.42 (0.96-5.99)	<1%	<1%	3%	97%	85%	66%
≥84°	3.04 (1.46-6.51)	<1%	<1%	<1%	>99%	97%	87%

* OR = odds ratio, CrI = credible interval, VA = variable angle, LCP = locking compression plate, LISS = less invasive stabilization system, DF = distal femur, LDFA = lateral distal femoral angle. The model was adjusted for a time coefficient (the time in days between the index surgery for each patient and the first surgical date). CrIs represent the range of values within which the OR lies with 95% probability.

† BMI of ≥30 kg/m^2^.

### Sensitivity Analysis

When including patients with ≥5.5 months of follow-up (n = 479), the evidence of an association between obesity and nonunion increased to very strong (pOR >1: from 93% to 96%) and the evidence of an association between multifragmentary metaphyseal comminution and nonunion (pOR >1: 96% to 95%) and between an LDFA of ≤78° and nonunion (pOR >1: 97% to 95%) decreased to strong (see Appendix 2). Using alternative categories for proximal screw density, screw densities of ≤0.50, 0.51 to 0.75, and 0.76 to 0.99 proximal to the working length were associated with reduced odds of nonunion (pOR <1: 98%, 92%, and >99%, respectively) when compared with a screw density of 1.00, whereas the evidence of a protective effect with the use of titanium plates increased from moderate to strong (pOR <1: 87% to 91%) (see Appendix 3).

## Discussion

This study aimed to identify and interpret risk factors for nonunion following lateral locked plating of distal femoral fractures with use of Bayesian analysis. Overall, 1 in 10 patients developed nonunion and underwent reoperation to promote healing. There was very strong evidence that multifragmentary metaphyseal fracture patterns, a moderate length of medial cortical comminution, and varus or valgus malalignment were associated with increased odds of nonunion. A screw density of ≤0.60 proximal to the working length was associated with reduced odds of nonunion; this finding was consistent with the sensitivity analysis results, although the size and certainty of the effect varied. There was strong evidence that obesity was associated with increased odds of nonunion and that intact wedge fractures were associated with reduced odds. Overall, the effect sizes of factors associated with nonunion were small to moderate.

The present study’s nonunion rate of 9.6% is within the range of previously published studies, which have reported rates ranging from 6% to 20%^2-11^. Augmented fixation constructs (e.g., nail-plate or dual-plate) are increasingly being used to obtain more reliable outcomes, with several studies demonstrating favorable results^25-27^. However, there is no definitive consensus on the indications for specific fixation constructs. Understanding the factors associated with nonunion following lateral locked plating can optimize treatment strategies and identify patients who may benefit from augmented fixation constructs.

The present study found a 96% probability that multifragmentary metaphyseal fracture patterns increased the odds of nonunion. Comminution has been identified as a risk factor in previous studies, although the definitions varied considerably^2,5,8^. Interestingly, the present study demonstrated a 90% probability that intact wedge fractures were protective against nonunion. This finding should be interpreted with caution given that the nonunion rates were low in both groups (simple fractures: 5 of 139 [3.6%]; intact wedge fractures: 2 of 71 [2.8%]) and that the distinction between simple and (nondisplaced) wedge fractures may not always be evident in clinical practice. Additionally, the results indicated a 96% probability that a moderate length (>0 to 25 mm) of medial cortical comminution was associated with a >1.5-fold increase in the odds of nonunion. Paradoxically, more extensive comminution (≥26 mm) did not demonstrate a similar effect, which may have been influenced by mediating or confounding variables (e.g., differences in postoperative rehabilitation strategies or patient demographics among those with more severe comminution). Additionally, research has shown that the morphology and alignment of the comminuted segment also contribute to medial column support and subsequent nonunion risk^5,11^. It may be helpful for future studies to establish consistent and reliable parameters for medial column integrity on the basis of both fracture and reduction parameters^28^. Overall, the results of the present study suggest that surgeons may consider augmenting fixation constructs on the basis of the presence of comminution and the loss of medial column integrity.

There was a 98% probability that a proximal screw density of ≤0.60 was associated with reduced odds of nonunion compared with a density of ≥0.81, whereas densities of 0.61 to 0.80 only had a 77% probability of the existence of this effect. In the sensitivity analysis, all density categories of <1.00 were associated with reduced odds of nonunion compared with a density of 1.00, albeit with varying size and certainty of effects. This suggests that filling all screw holes proximal to the working length should be avoided. Such a configuration reflects an era in which relatively short plates were filled with unicortical locking screws, which may have resulted in rigid constructs that suppressed callus formation^2,29^. Translating findings from density values of <1.00 to clinical practice is less straightforward because there was no linear association between screw density and nonunion risk and because screw density can be modulated by the number, distribution, and location of proximal screws, as well as by the proximal plate length. Using a finite element analysis of a distal femoral fracture with a 50-mm metadiaphyseal gap, Erbulut et al. showed that distributing proximal screws over a longer plate length reduced the risk of pull-out^30^. This finding is relevant given the likelihood of poor bone quality in the present study population (median age, 68 years; 71% female). Interestingly, other factors that have previously been associated with construct biomechanics—most importantly, working length and proximal screw modality^31-35^—did not show evidence of an association with nonunion in the present study. When comparing the present study with clinical studies, the current study findings seem to contrast with those of Stockton et al., who reported that increased screw density was protective against nonunion^11^. However, their definition of screw density used the total number of plate holes proximal to the fracture as the denominator. This difference likely contributed to the different screw densities (the IQR for screw densities as a continuous variable was 0.57 to 0.88 in the present study versus 0.30 to 0.50 in the study by Stockton et al.) and limits comparisons. Other studies have reported inconsistent results regarding the effect of screw density on nonunion^2,4,12,13^. Considering the inconclusive evidence, and given that construct characteristics are inherently related, readers are encouraged to interpret the results in the context of patient, fracture, and treatment characteristics rather than as absolute findings.

The present study found strong evidence that coronal plane malalignment contributed to nonunion, with an LDFA of ≥84° associated with a 3.0-fold increase and an LDFA of ≤78° associated with a 2.4-fold increase in the odds of nonunion. Similarly, Stockton et al. found that an LDFA of ≥84° was associated with a twofold increase in the odds of nonunion^11^. Malalignment in the coronal plane is common after lateral locked plating of distal femoral fractures, and studies have suggested that implant design and position may contribute to this^1,10,13,36^. Medial translation of the condylar block (“golf club deformity”) is another reduction parameter in the coronal plane that has been associated with nonunion^5,11^. However, in the present study, there was only weak evidence that this variable increased the odds of nonunion. This may reflect influence from similar factors that also contributed to the variability observed across different lengths of medial cortical comminution. Nevertheless, surgeons should be cognizant of coronal plane alignment in order to optimize the healing of distal femoral fractures following lateral locked plating.

The only patient characteristic with strong evidence of an association with increased odds of nonunion was obesity. This finding is consistent with previous studies that identified increased BMI as a risk factor for nonunion^3-5,9^. The effect of BMI on the failure of treatment involving lateral locked plating may stem from an impaired biological response to fracture-healing, increased surgical difficulty resulting in suboptimal fixation, and additional stress on the fixation construct^5,37,38^. Surgeons may therefore consider augmenting fixation constructs to better withstand mechanical stresses while minimizing biological insult. Nevertheless, it is important to consider that the effect of obesity seemed to be modest, with weak evidence that the increase in the odds of nonunion exceeded 50%.

### Limitations

The present study has limitations. First, this retrospective study spans an 18-year period, during which implants and treatment strategies have evolved. Nevertheless, lateral locked plating was the standard-of-care treatment for distal femoral fractures during the study period, and the final models included multiple explanatory variables, which strengthens the findings. Second, a follow-up of ≥3 months was chosen to limit the effect from patients who did not experience healing complications and were lost to follow-up beyond 3 months. A similar approach has been used in previously published studies^5,11^. The findings from the sensitivity analysis that included patients with ≥5.5 months of follow-up were consistent with the primary analysis, with minor differences in evidence. Third, there are no accepted thresholds to guide the interpretation of probability estimates derived from Bayesian analysis. The present study defined probabilities of >95% as very strong evidence. However, estimates of ≤95% were also reported, given that a dichotomous interpretation and reporting of effects (i.e., absent versus present) may not always acknowledge relationships where one exists. As such, compared with the frequentist approach, Bayesian analysis better aligns with the reasoning utilized in clinical practice (see Appendix 1). Fourth, the study population size was modest and may represent distinct clinical populations. Future studies may benefit from stratified analyses of more homogeneous subgroups. In addition, the majority of patients were White and non-Hispanic, which may affect generalizability. Fifth, nonunion was defined as a reoperation to promote healing, which may have excluded patients with radiographic evidence of nonunion who did not undergo reoperation. However, there is no consensus on the radiographic criteria for the healing of distal femoral fractures, and the definition in the present study aligns with that utilized in previously published studies, thereby aiding comparability across studies^5,11,39^. Lastly, the study relied on radiographic measurements, which may be subject to measurement errors and may not fully capture the 3-dimensional nature of fracture patterns. However, the lower bounds of the 95% CIs of the ICCs indicated at least good reliability for medial translation and the LDFA and excellent reliability for medial comminution, supporting reproducibility^40^.

### Conclusions

In this study of 560 patients undergoing lateral locked plating for a distal femoral fracture, 1 in 10 patients developed nonunion and underwent reoperation to promote healing. There was very strong evidence that multifragmentary metaphyseal comminution, a moderate length (>0 to 25 mm) of medial cortical comminution, and coronal plane malalignment increased the odds of nonunion. Surgeons should therefore aim to restore coronal plane alignment and may consider augmenting fixation in the presence of multifragmentary comminution. A screw density of ≤0.60 proximal to the working length reduced the odds of nonunion, although the size and certainty of this effect varied in the sensitivity analysis that utilized alternative screw density thresholds. Constructs in which all screw holes proximal to the working length are filled should be avoided, but the optimal construct configuration remains unclear and depends on other construct characteristics. Overall, the small to moderate effect sizes highlight the multifactorial etiology of nonunion.

## Appendix

Supporting material provided by the authors is posted with the online version of this article as a data supplement at jbjs.org (http://links.lww.com/JBJS/I944).
